# Marshes as “Mountain Tops”: Genetic Analyses of the Critically Endangered São Paulo Marsh Antwren (Aves: Thamnophilidae)

**DOI:** 10.1371/journal.pone.0140145

**Published:** 2015-10-08

**Authors:** Crisley de Camargo, H. Lisle Gibbs, Mariellen C. Costa, Glaucia Del-Rio, Luís F. Silveira, Adriane P. Wasko, Mercival R. Francisco

**Affiliations:** 1 Departamento de Genética, Instituto de Biociências, Universidade Estadual Paulista “Júlio de Mesquita Filho”, Distrito de Rubião Júnior, s/n, CEP 18618–970, Botucatu, São Paulo, Brazil; 2 Department of Evolution, Ecology and Organismal Biology, Ohio State University, Columbus, Ohio 43210–1293, United States of America; 3 Departamento de Ciências Ambientais, Universidade Federal de São Carlos, Campus de Sorocaba, Rod. João Leme dos Santos, km 110, CEP 18052–780, Sorocaba, São Paulo, Brazil; 4 Museum of Natural Science, Louisiana State University, Baton Rouge, Louisiana 70803, United States of America; 5 Seção de Aves, Museu de Zoologia da Universidade de São Paulo, Caixa Postal 42494, CEP 04218–970, São Paulo, São Paulo, Brazil; Australian National University, AUSTRALIA

## Abstract

Small populations of endangered species can be impacted by genetic processes such as drift and inbreeding that reduce population viability. As such, conservation genetic analyses that assess population levels of genetic variation and levels of gene flow can provide important information for managing threatened species. The São Paulo Marsh Antwren (*Formicivora paludicola*) is a recently-described and critically endangered bird from São Paulo State (Brazil) whose total estimated population is around 250–300 individuals, distributed in only 15 isolated marshes around São Paulo metropolitan region. We used microsatellite DNA markers to estimate the population genetic characteristics of the three largest remaining populations of this species all within 60 km of each other. We detected a high and significant genetic structure between all populations (overall *F*_ST_ = 0.103) which is comparable to the highest levels of differentiation ever documented for birds, (e.g., endangered birds found in isolated populations on the tops of African mountains), but also evidence for first-generation immigrants, likely from small local unsampled populations. Effective population sizes were small (between 28.8–99.9 individuals) yet there are high levels of genetic variability within populations and no evidence for inbreeding. Conservation implications of this work are that the high levels of genetic structure suggests that translocations between populations need to be carefully considered in light of possible local adaptation and that remaining populations of these birds should be managed as conservation units that contain both main populations studied here but also small outlying populations which may be a source of immigrants.

## Introduction

Endangered species often exist in small isolated populations, which can suffer increased risks of extinction due to inbreeding and loss of adaptive genetic variation through genetic drift [1–3]. The degree to which these genetic mechanisms have negative impacts on population viability is influenced by levels of gene exchange between populations because gene flow can maintain levels of genetic variability within populations and counteract the effects of drift [4–6]. Thus, measuring the degree of genetic isolation between small populations of endangered species is important to assess if these genetic risks are present for a given species. The degree of genetic differentiation between populations can also influence the choice of management strategies to counter the possible negative genetic consequences of drift in small populations. In particular, if populations range in size, the translocation of individuals from the larger populations can be used to raise the level of genetic variability in the smaller ones [7–10]. When population differentiation is low, there are no negative genetic consequences for this kind of management [8], but when populations are structured translocations may not be desirable due to the risks of disrupting local adaptation [7, 9, 11–13]. For these reasons, measuring the genetic structure, levels of genetic variability, and amounts of gene flow between existing populations is pivotal to the conservation and management of endangered species in human impacted landscapes [3, 8, 14, 15].

One species where such an analyses would be especially useful is the São Paulo Marsh Antwren (*Formicivora paludicola*, Thamnophilidae). This endangered species is a small insectivorous bird only recently described as a distinct species [16]. It is endemic to the Atlantic Forest of São Paulo State, Brazil, with a total population estimated to be no more than 250–300 individuals. Exhaustive searches from 2006 to 2010 located 15 populations all in isolated marshland fragments around the metropolitan region of São Paulo city, in Tietê and Paraíba do Sul river Basins [16], but a recent study revealed that the species is now extinct in two of these areas [17]. Due to the limited population size, and its restricted distribution, this species has been classified as “Critically Endangered” in Brazilian Red List [18], and may be included in global lists with a similar classification in the near future. Ongoing threats for existing populations include marsh degradation due to sand mining, drainage for pasture and agriculture, fire, and invasion by exotic grasses [16, 19]. These birds currently exist in small isolated marsh areas all less than 45 ha implying small population sizes with a high potential for drift.

Other population genetic studies of tropical birds have shown that most species have limited levels of genetic structure [20–22] although there are exceptions [14, 23]. One reason why these antwrens could show high levels of genetic structure is that they are wetland-specialists. Wetlands are typically patchy environments imbedded in a matrix of upland habitats [24, 25]. As a consequence, resident marsh-dependent species exist in small and isolated populations that are connected through occasional migration, forming metapopulations which may show high degrees of genetic structure due to the combined effects of drift in small populations and limited gene flow [25, 26]. This prediction has been supported through studies showing high levels of genetic structure in various marsh-dependent organisms [27, 28].

To assess the genetic characteristics of São Paulo Marsh Antwren we used microsatellite DNA loci to analyze the genetic variability of the three largest remaining populations of this bird. Our goals were to: 1) Measure levels of genetic variability within and genetic differentiation between antwren populations located in Mogi das Cruzes, Salesópolis, and São José dos Campos, 2) Estimate genetically effective population sizes and assess if populations have experienced recent bottlenecks, and 3) Assess levels of population connectivity through the detection of first generation migrants. Our overall goal is to use genetic information to evaluate if this species potentially faces present-day or future genetic risks that may impact its long-term viability and which mechanisms operate to influence levels of variation in the few remaining populations of this highly endangered bird.

## Materials and Methods

### Study species

**Fig 1:**
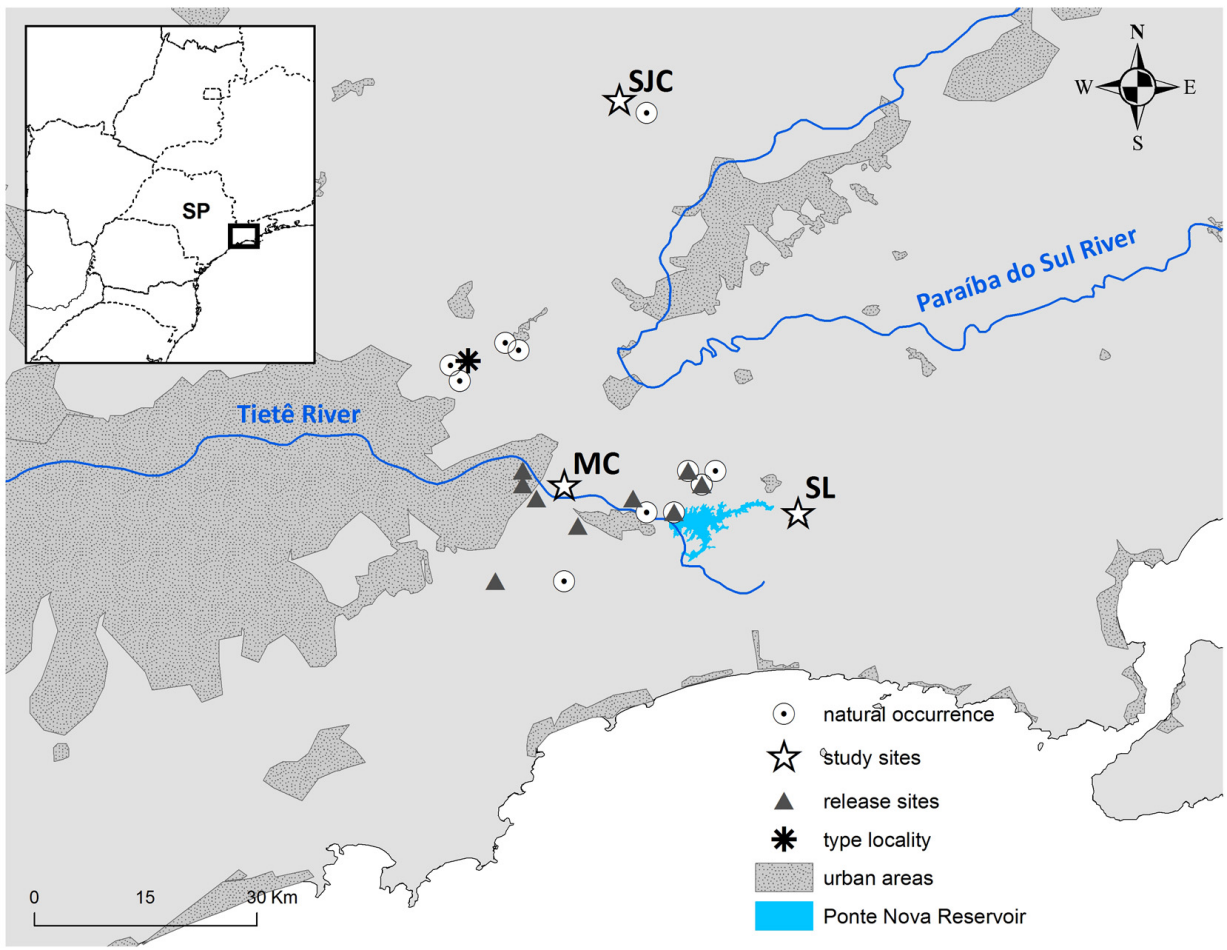
São Paulo Marsh Antwren distribution. Locations where the São Paulo Marsh Antwren has been found along with release sites near the metropolitan region of the city of São Paulo. Letters indicate our study sites where samples were collected: São José dos Campos (SJC), Mogi das Cruzes (MC), and Salesópolis (SL). These represent the largest extant populations. The map was produced in Quantum GIS 2.2 [29]. The urban areas and reservoir contours were obtained by LANDSAT 8 imagery [30].

São Paulo Marsh Antwren was first discovered in 2004 [16]. It is a small (10 g), sedentary, secretive bird that lives under tall dense marsh vegetation [16]. Exhaustive searches were performed to locate the existing populations of this species after its initial discovery and it proved to have a restricted distribution, limited to only 15 marsh patches (all < 45 ha) in the headwaters of Tietê and Paraíba do Sul rivers, from 600 to 760 m above sea level in the municipalities of Biritiba-Mirim, Mogi das Cruzes, Salesópolis, Santa Isabel, and São José dos Campos, in São Paulo State, southeastern Brazil (Fig 1) [16, 17, 29, 30]. In 2005, a population from Biritiba-Mirim was threatened with flooding by the construction of a dam in Tietê River (Barragem do Paraitinga). As a result 72 individuals were captured in this area and were released in 10 nearby marshes in which the species was not previously observed (Fig 1). In a search carried out in 2008 most of these birds were still present at the release sites, where juveniles were also observed, indicating successful breeding of relocated birds [16].

### Study sites

We sampled birds at the three major marshes in which this species occurs (Fig 1). Two of the areas are located in Upper Tietê basin: Mogi das Cruzes (MC) (23°32’S, 46°07’W, around 45 ha in area), and Salesópolis (SL) (23°34’S, 45°49’W, around 30 ha). These areas are on separate sides of the Barragem do Paraitinga reservoir, which has a total flooded area of 6,437 km^2^. The third area is São José dos Campos (SJC) (23°04’S, 46°02’W, around 20 ha), located in Upper Paraíba do Sul basin. These marshes are surrounded by a mosaic composed mainly of *Eucalyptus* spp. silviculture, pasturelands, and Atlantic Forest fragments. The linear distances between these areas vary from 30 to 60 km. The other marshes in which this species occur are all smaller than 11 ha and probably contain small numbers of individuals (G Del-Rio, unpublished data).

### Bird sampling and DNA extraction

São Paulo Marsh Antwrens live in pairs that defend small territories all year round which are easily identifiable in the field, as these birds promptly respond to playbacks of their song. At each study site, we first used playbacks to locate the territories. Once a territory was identified, we set up a mist net of 12 × 2.5 m, and playbacks were used again to attract the birds to the net. All captured birds were banded using a unique combination of PVC colored rings for individual identification, and a 10–20 μL blood sample was obtained from each bird by cutting the tip of a nail, after which birds were released [22]. Blood was then mixed with an amount of 0.5 M EDTA, and this was immediately added to a 1.5 mL tube containing 100% ethanol. After returning from the field, samples were stored in a -20°C freezer.

Using these techniques, we collected blood samples from 57 birds from 2012 to 2014 (Table 1). Although sample sizes are small, the numbers collected at each site are proportional to the size of each area. Except for MC, we have captured at least one member of all of the pairs that were defending territories in these areas. Bird capture and blood sampling methods were approved and authorized by the responsible Brazilian Federal Government institution (Ministério do Meio Ambiente, Instituto Chico Mendes de Conservação da Biodiversidade, SISBIO/ICMBio: permit #36562–1). Study sites were private lands, and their owners (Suzano Papel e Celulose, and other private individuals) permitted access to each area.

**Table 1:** Number of analyzed individuals (*N*), average number of alleles per loci (*N*_A_), allelic richness (*A*_R_), observed (*H*_O_) and expected (*H*_E_) heterozygosities, inbreeding coefficient (*F*_IS_), and its probability of being different from zero (*P*), across 17 microsatellite loci, found in three populations of the São Paulo Marsh Antwren. The critical P value after Bonferroni correction was 0.001. (SD: Standard Deviation).

Population	*N*	*N*_A_ (SD)	*A*_R_ (SD)	*H*_O_ (SD)	*H*_E_ (SD)	*F*_IS_	*P*
Mogi das Cruzes	26	6.00 (± 2.00)	5.35 (± 1.55)	0.70 (± 0.14)	0.71 (± 0.13)	0.020	0.233
Salesópolis	17	4.76 (± 1.39)	4.55 (± 1.23)	0.66 (± 0.14)	0.66 (± 0.09)	0.001	0.493
São José dos Campos	14	5.77 (± 1.60)	5.69 (± 1.56)	0.70 (± 0.16)	0.73 (± 0.10)	0.037	0.176

### Microsatellite genotyping

DNA was extracted from blood using a standard phenol-chloroform-isoamyl alcohol protocol [31]. For each individual, DNA fragments were amplified for 9 species-specific microsatellite loci (Fpa11, Fpa13, Fpa14, Fpa15, Fpa17, Fpa18, Fpa23, Fpa24, and Fpa25 –[32]), and 8 heterologous loci developed for Chestnut-backed Antbird (*Myrmeciza exsul*) (MyEx19, MyEx41, MyEx46, Mex034, Mex120, Mex140, Mex162, and Mex176 –[33, 34]), using fluorescently labelled primers. PCR reactions details and amplification conditions are described in [32]. Amplified products were run on an ABI 3500 sequencer, and allele sizes scored using software GeneMarker2.4.0 (Softgenetics). Raw microsatellite genotypic data is presented in S1 File.

### Population differentiation

Population structure was inferred using the Bayesian method implemented in Structure 2.3.4. [35]. We used an admixture model with correlated allele frequencies, and default parameter settings. We ran 1,000,000 MCMC iterations (discarding 10,000 as burn-in), and ran five replicates of each *K* (from *K* = 1 to *K* = 6). The most appropriate *K* was defined using the Evanno method [36], implemented in the software Structure Harvester [37]. The levels of genetic differentiation between the populations were estimated by three different methods. First we calculated the Fixation Index (*F*_ST_) [38], and assessed its significance by testing if genotypic distribution was identical between populations using the log-likelihood (G) based Exact Test, implemented in Fstat 2.9.3.2 [39], after 10,000 permutations. Second, we estimated the *F*’_ST_ of [40]. These authors have demonstrated by simulations that in a metapopulation scenario levels of population differentiation using some of the traditional metrics can be underestimated if the number of sampled populations is small, an effect that also can be caused by the high levels of within-population heterozygosity promoted by the use of highly variable microsatellites (for a review, see [40]). The corrected *F*’_ST_ is unaffected by this sampling bias and incorporates a correction for within-population genetic diversity bias. Its significance was tested using the AMOVA procedure in GenAlEx 6.5 [41, 42], with 10,000 permutations. Third, we used the software SPAGeDi1-5a [43] to estimate *R*_ST_ [44], with significance estimated using 10,000 permutations of individuals between pairs of populations.

Finally, to gain insight into the degree to which levels of differentiation reflect long versus short-term isolation, we performed the *R*_ST_ allele size randomization test of [45] as implemented in the software SPAGeDi1-5a, using 10,000 permutations. In this analysis, allele sizes are permuted among allelic states to assess whether stepwise mutations have made significant contribution to the observed levels of genetic differentiation. Significant results imply that alleles that originate from novel mutations arising over evolutionary timescales have had a significant impact on levels of differentiation hence that populations have been isolated for long periods of time. Nonsignificant results suggest that frequency differences in existing alleles that have developed over more recent timescales can alone account for observed levels of genetic structure and indicate that the observed differentiation has developed more recently [45, 46].

### Levels of genetic variation

We estimated observed (*H*_O_) and expected (*H*_E_) heterozygosities using Genepop 4.2 [47]. Genetic diversity within each population was measured by calculating allelic richness (*A*_R_) scaled for differences in sample size between populations [48] also using FSTAT. Finally, we assessed levels of inbreeding by calculating the inbreeding coefficient (*F*_IS_) [38] for each locus, and then across all loci using Fstat [39]. To evaluate whether these values were significantly different from zero, we used a permutation procedure.

### Identifying first generation immigrants

To evaluate levels of contemporary immigration, we used assignment tests in GeneClass2 [49] to detect first-generation immigrants in each population by estimating the probability of an individual’s multilocus genotype to belong to the population in which it has been sampled (L_home likelihood). This procedure is indicated when unsampled populations could serve as source of immigrants [49, 50]. Likelihood values were obtained by the frequencies-based method of [51], and probabilities were estimated using the algorithm of [50], with 1,000 Monte Carlo resamplings and a critical value (α) of 0.05. The probability of excluded individuals being assigned to other populations in the analyses was then evaluated using a similar conceptual approach.

### Effective population size and recent bottlenecks

Effective population size was accessed using the linkage disequilibrium method implemented by software NeEstimator, Ver. 2 [52], using the Monogamy mating option—inferred by the reproduction system of its sister-species, Marsh Antwren (*F*. *acutirostris*) [53]–and a critical value of 0.02 to discard rare alleles. We evaluated possible recent bottlenecks using the Two Phase Model (TPM) [54] in Bottleneck [55, 56]. Microsatellites have been shown to evolve mainly through single-steps mutations, but rare multi-step changes may also occur and microsatellite mutational models must take this into account [54]. In TPM model the percentage of pure stepwise mutations is specified, and the number of mutational steps of multi-step changes is drawn from a geometric distribution, with specified variance [54]. As about 60 to 80% of avian microsatellite mutations involve single-step changes [28], we used an intermediate value of 70%. To evaluate the consistency of our results, we used the range of variances proposed by [28] (4, 9, 16, and 25) to parameterize the distribution. These values correspond approximately to 2, 3, 4, and 5 mutational steps, respectively [28, 54]. Significance was estimated using Wilcoxon signed-rank test, which performs better when less than 20 loci are used [56], with 10,000 permutations.

## Results

**Fig 2:**
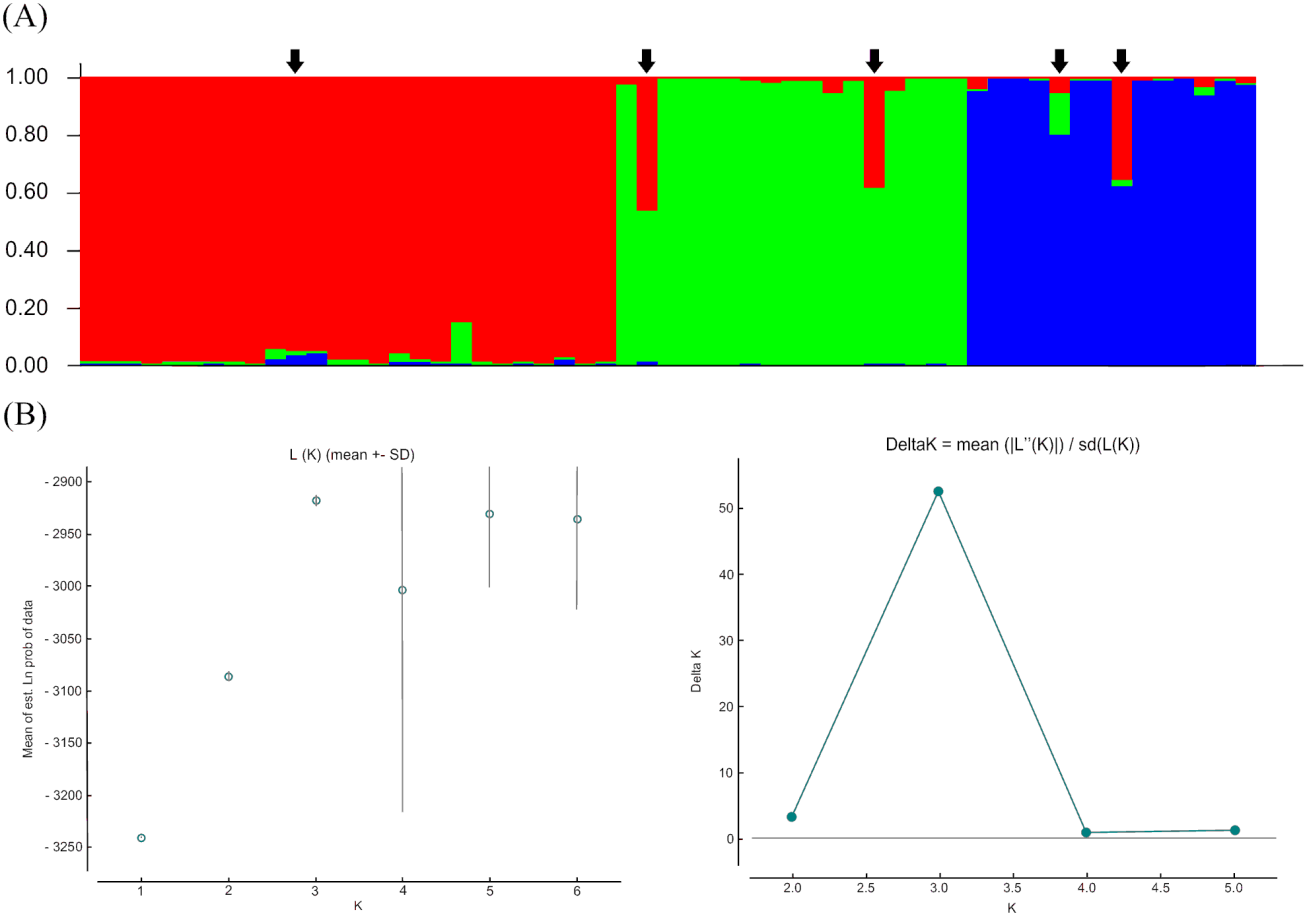
Population structure and first generation migrants. **(A)** Proportional membership (*Q*) of each São Paulo Marsh Antwren in the genetic clusters inferred by Structure (*K* = 3). This graphic represents each individual as a bar, and its membership probability in each cluster. Individuals **1** to **26**: Mogi das Cruzes population; Individuals **27** to **43**: Salesópolis population, and individuals **44** to **57:** São José dos Campos. Black arrows identify individuals indicated as first generation migrants. **(B)** Output graphics from Structure Harvester, indicating the higher value of *Ln P*(*K*) (left), and Δ*K* (right).

The Bayesian analyses run in Structure indicated K = 3 as the most appropriated number of genetic clusters (K) for the sample and these clusters mirror our three sample sites exactly, although there is evidence for a small number of admixed individuals consistent with the presence of immigrants (see below) (Fig 2). Consistent with this result, *F*_ST_, *F*′_ST_, and *R*_ST_ values between the three sites were all high and all highly significantly different from zero (Table 2). Allele size randomization procedure did not reveal significant stepwise mutation influence in the levels of population divergence, being P = 0.664 for Mogi x São José dos Campos; P = 0.207 for São José dos Campos x Salesópolis, and P = 0.097 for Mogi x Salesópolis suggesting that the differentiation between populations is primarily the result of shifts in the frequency of existing alleles and not the origin of novel alleles through mutations.

**Table 2:** *F*_ST_ and *F′*_ST_ (in parentheses) values between pairs of studied populations (above diagonal), and *R*_ST_ values (bellow diagonal). The overall *F*_ST_, *F′*_ST_, and *R*_ST_ values across all populations were 0.103, 0.344, and 0.097, respectively. All P values are < 0.01.

Population	Mogi das Cruzes	Salesópolis	São José dos Campos
Mogi das Cruzes	-	0.101 (0.324)	0.098 (0.349)
Salesópolis	0.136	-	0.116 (0.375)
São José dos Campos	0.047	0.165	-

Despite the high level of genetic differentiation, assignment test results from GeneClass suggest the presence of first generation immigrants in each population: one first generation immigrant was detected in MC, two in SL, and two in SJC (P < 0.05 –black arrows in Fig 1). Thus, first generation immigrants make up between 4–14% of the birds in each population. While these identified immigrants match the admixed individuals in the Structure analysis, in fact, the GeneClass results exclude these individuals as originating from one of the three sampled populations as they fail to assign them to any of the sampled populations.

Even though they are isolated, levels of genetic variation in these populations are high and there is no evidence for inbreeding. Observed (*H*_O_) and expected (*H*_E_) heterozygosities averaged respectively 0.70 (range: 0.35–0.89) and 0.71 (range: 0.36–0.87) for MC, 0.66 (range: 0.41–0.88) and 0.66 (range: 0.50–0.82) for SL, and 0.70 (range: 0.36–0.86) and 0.73 (range: 0.43–0.90) for SJC (Table 1 and S1 Table). Consistent with the similarity in *H*_O_ and *H*_E_ values, *F*_IS_ values showed no significant divergence from zero in all of studied populations (Table 1) providing no evidence for inbreeding. The average allelic richness (*A*_R_) was similar across populations: 5.35 alleles (range: 2.88–8.69) for MC, 4.55 alleles (range: 2.00–7.28) for SL, and 5.69 alleles (range: 3.00–9.85) for SJC. The estimated effective population size was 99.9 individuals (95% CI: 69.5–165.8) for MC, 28.8 individuals (95% CI: 21.5–40.7) for SL, and 34.4 individuals (95% CI: 25.3–50.4) for SJC.

Finally, only one population shows evidence for recent population decline. Except for one marginal probability (P = 0.080), Bottleneck revealed highly significant heterozygosity excess for Mogi das Cruzes (MC) across all of the variance values used in the distribution of mutational steps, suggesting that a recent bottleneck may have occurred in this population, but it did not show the occurrence of recent bottlenecks neither in Salesópolis (SL) nor in São José dos Campos (SJC) (Table 3).

**Table 3:** Probabilities of recent population decline obtained in BOTTLENECK for the three main populations of São Paulo Marsh Antwren across a range of variance values (4, 9, 16, and 25) used to parameterize the distribution of multi-step microsatellite mutations.

Population	4	9	16	25
Mogi das Cruzes	0.080	0.011	0.006	0.003
Salesópolis	0.306	0.274	0.189	0.142
São José dos Campos	0.339	0.258	0.215	0.164

## Discussion

### Population genetic structure

One of the most striking results of this study are the high levels of genetic structure that are detected over a limited geographic scale between the three largest remaining populations of this critically endangered bird. In particular, the observed *F*_ST_ values were substantially higher than those documented for other populations of tropical forest-dependent passerine birds, from either continuous or fragmented habitats studied in similar geographic scale [20, 21, 22]. Most relevant are comparisons with the levels of differentiation between populations of Chestnut-backed Antbird a related forest understory species that also belong to the family Thamnophilidae [21]. Populations of this species from forest fragments isolated for 60 years showed microsatellite-based *F*_ST_ values that were one order of magnitude lower than those observed here [21]. One key difference between São Paulo Marsh Antwren and Chestnut-backed Antbird that might explain this difference is habitat use. Antbirds are generally forest specialists whereas the São Paulo Marsh Antwren only breeds in wetland marshes. Wetlands are patchily distributed throughout the landscape which might lead to higher levels of genetic structure in marsh specialist species due to increased isolation [24–26]. However, other marsh-specialist birds also show levels of genetic structure less than that we observed. For example, North American populations of the Black Rail (*Laterallus jamaicensis*) showed microsatellite-based *F*_ST_ estimates of 0.03 between populations in marshes that were a comparable distance (160 km) apart, with *F*_ST_ values of 0.1 only observed for geographically widely-separated populations ≥ 1100 km apart [27]. Similarly, in another marsh species, the Yellow Rail, (*Coturnicops noveboracensis*) *F*_ST_ values of 0.1 were found for populations that were 2,000 to 3,800 km apart [28]. Our conclusion is that habit use of wetlands alone cannot explain the high structure shown in the antwrens (see below).

Our high *F*_ST_ over small geographic scale are most comparable to differentiation documented by [14] for populations of endangered Taita Thrush (*Turdus helleri*), Yellow-throated Woodland-warbler (*Phylloscopus ruficapilla*), and Stripe-cheeked Greenbul (*Andropadus milanjensis*) found in cloud forest patches < 20 km apart on the top of mountains which are isolated by lowland savannas and degraded habitats in Tanzania [14]. In particular, most comparable to São Paulo Marsh Antwren is the Taita Thrush. This is a critically endangered bird which is hypothesized to show high genetic structure due to the extremely low dispersal ability as confirmed from radio-telemetric and mark-recapture data [14, 57], and the natural isolation of the forest patches in which it lives in the top of the mountains combined with habitat fragmentation in intervening habitats that may have started several hundred years ago [14].

High values obtained for *F′*_ST_ were expected as we have used highly polymorphic microsatellites, and at a local scale each of the São Paulo Marsh Antwren populations studied here is likely part of a set of metapopulations (see below). Our observed levels of *F*’_ST_ were higher than those obtained for populations of Réunion Grey White-eye, *Zosterops borbonicus* (0.12 to 0.21) that were 8.8 to 25.2 km apart. This is a passerine bird endemic to Réunion Island for which gene flow between populations is extremely low, probably due to behavioral processes [58]. Finally, *R*_ST_ estimates were high and comparable to populations of cloud forest specialist passerines isolated in mountain tops. For example, in the East African Mountain White-eye, *Zosterops poliogaster silvanus*, *R*_ST_ values varied from 0.098 to 0.243 between populations approximately 15 to 80 km apart [23]. However, the allele size permutation test of [45] was not significant for any comparison which suggests that the differentiation between populations is primarily the result of frequency differences in existing alleles and not the origin and increases in frequency of novel alleles originated through stepwise mutations [45, 46].

One striking result that is inconsistent with our observation of high levels of genetic differentiation is the relatively large portion of first generation migrants (4–14%) in each population. This high level of immigration suggests the potential for high levels of gene flow leading to less genetic structure than is currently observed assuming that these individuals could successfully breed. The fact that we were unable to assign these immigrants to sampled populations suggests that in fact, these birds do not represent potential genetic migrants between the main populations but are rather from local sources near each of the main populations.

We see three possible scenarios for the origin of these immigrants. Under the first scenario these individuals are birds that were relocated during the establishment of the reservoir who did not settle in the new areas but moved into our study populations. However, this is unlikely because all birds that were translocated were banded and all the individuals identified as immigrants were unmarked. Under the second scenario the immigrant birds represent an unusual one-time event in which individuals from unknown populations existing in the flooded regions were displaced and moved into the Salesópolis and Mogi study areas, which are located on different sides of the reservoir. However, given the limited dispersal ability of São Paulo Marsh Antwren, this mechanism cannot explain the presence of immigrants in São José dos Campos, which is in a separate river (Paraíba do Sul) basin, to which dispersal from the reservoir region would be unlikely. The third, and in our view most plausible scenario, is based on the fact that surveys have shown that there are additional small populations consisting of limited numbers of individuals around each of the three main populations [16] (see Fig 1) that were not included in our sampling. Our hypothesis is that each of our currently defined “populations” actually consists of a single large (sampled) population combined with an unknown number of unsampled small populations, which in Salesópolis and Mogi could include later generations of translocated populations, and that these sets of one bigger and multiple smaller populations form metapopulation complexes [59]. Movement of birds between the large and the small populations within each complex represents the source of our observed immigrants and may represent an important source of genetic replenishment for the large populations over historical timescales (see below). However, movement between the three large complexes centered around the sampled populations is limited and this accounts for the high levels of genetic structure that we observed. This hypothesis could be tested through more detailed sampling and assessment of patterns of movement by birds from the small local populations next to each of the large populations included in this study.

Our analyses of population characteristics argue that these antwrens have likely existed in small isolated populations for substantial periods of time. The lack of evolutionary signal in the allele sizes randomization test [45] indicates this time scale is less than one where mutational processes affecting the origin and increase in microsatellite allele frequencies have significant effects on population variation. Regardless of the specific timescale, our findings suggest that the recent evolutionary history of these birds has been shaped by their patchily distributed wetland environment on which they depend similar to the mountain top dependent species in Africa studied by [14]. Other related species of Formicariidae that inhabit forest understory habitats also have adaptations to these environments that make them unwilling to cross open habitats and hence are poor colonists [59, 60] leading to high levels of population structure [21, 61, 62]. For the São Paulo Marsh Antwren, adaptations to survive in the midst of tall cattails, an environment that like the dense forest understory does not permit long flights, may have led them to evolve a similar inability to disperse. However, for this species, continuous forest habitat may have also acted as a barrier to dispersal. For a sister species, the Marsh Antwren, that inhabits tidal marshes from southern Brazil, Reinert et al. [63] obtained experimental evidence that individuals cannot fly more than 25 m without landing, and they are unwilling to cross open areas. Before the habitat fragmentation occurred in the last 150 years in our study region [64], the marshes of Salesópolis and Mogi, both in the same river basin, were probably connected by a chain of forest streams, but the historical isolation we observed suggests a limitation of this species to follow streams inside the forest to disperse to other marshes. This extreme adaptation to a naturally small and patchy environment probably has contributed to the rarity and hence vulnerability of this bird.

### Population sizes

Current effective population sizes of the three studied populations were small (~ 50 birds) and similar to those obtained for the Chestnut-backed Antbird in small and isolated forest fragments in Costa Rica [21], but are an order of magnitude lower than that found for another Atlantic Forest passerine, the Blue Manakin (*Chiroxiphia caudata*) [22]. Surprising, despite the small sizes of these populations we found no evidence for inbreeding which we attribute to low levels of gene flow into the sampled populations from nearby small populations. Based on a lack of evidence for bottlenecks in two of three populations this bird appears to have existed in small stable populations for substantial periods of time suggesting that they may not have suffered the effects of inbreeding depression. The recent bottleneck in the MC population may be evidence of impending effects of anthropogenic impacts, as apparently this area has been jeopardized by fires (MR Francisco, personal observation).

Frankham [65] observed that for socially monogamous passerine birds genetic effective population sizes varied from 28 to 74% of the census size. Assuming these extremes, we can provide a rough estimate of the total number of São Paulo Marsh Antwrens in these populations which represent the majority of extant individuals in this species. Applying these multipliers leads to a total population estimate of between 220 to 582 individuals. We estimate that in terms of area, the three populations we analyzed represent approximately 60% of the marshes in which this species occurs [16]. Our estimate suggests that there are more birds present than the preliminary census estimates provided by Buzzetti et al. [16] of 250 to 300 individuals in all of the 15 populations, but still confirms the status of this species as one of the most endangered birds in the Neotropics.

### Conservation implications

We see several conservation implications to our study. First, when populations are small and isolated, translocations of individuals between them has been suggested as a management choice to increase population viability [3, 7, 9, 10, 66], but here we have evidence that it may not be an appropriate strategy for maintaining the main São Paulo Marsh Antwren populations. High levels of population structure can be an indicative of local adaptation [7, 9, 11, 12]. Although our data do not allow us to assess if local adaptations are present, future work which analyzes whether fitness-related traits show population differentiation could provide valuable information for the management of this species. Meanwhile, not performing translocations would be a prudent management decision to avoid the potential disruption of local adaptations and outbreeding depression [7, 9, 13, 67]. Given a lack of detected inbreeding in these populations, translocations to increase genetic variability do not seem to be currently necessary.

Second, the low overall population numbers of this species and the potential importance of peripheral populations as sources of novel genetic variation mean that protection of all the 15 areas where São Paulo Marsh Antwrens are found should be a priority. This is especially important given that all of these sites are currently unprotected and exposed to human impacts [16]. As the creation of conservation units is often a costly and long-term process, founding new populations in marshes existing in areas within the species distribution that are already protected would be a potential short term alternative. Specific conservation units where this could be attempted are Parque Estadual Nascentes do Tietê and Área de Proteção Ambiental Banhado, both of which still retain significant remnants of marsh habitat. In this case, using individuals from all the major populations to maximize genetic variability could increase the chances of adaptation to these new areas [11, 68]. The vegetation structure is a potential source of significant habitat variation given that some areas are almost entirely dominated by cattails, *Typha domingensis* (Typhaceae), whereas in others variable proportions of Cyperaceae are present (e.g. *Schoenoplectus* sp., and *Rhynchospora* spp.) [16]. Most of the populations created through translocations during the creation of the reservoir in 2005 survived, although their long-term viability is not known. If future translocations occur then parameters such as effective population sizes, genetic variability of the source individuals [66, 69], and density of marshes in nearby areas, should be considered.

Our study demonstrates that populations of São Paulo Marsh Antwren distributed in marshes embedded within the Atlantic Forest can be highly isolated, generating genetic diversification in a level comparable to mountain-top bird populations in Africa (“marshes as mountain-tops”). But it is important to note that mountain tops may be more stable environments than marshlands. Marshes can naturally disappear soon [70], but the probability of new marshes being formed may be significantly reduced by anthropogenic landscape use. This draws attention to conservation of marshes in Atlantic Forest habitats that have often been neglected in conservation actions in this region. For instance, these habitats have not been included in mapping efforts, and consequently do not make part of estimates of habitat loss [71, 72]. Further studies are needed to assess whether this pattern of diversification and conservation concern could extend to other Atlantic Forest marsh—dependent organisms.

## Supporting Information

S1 File Raw microsatellite data for the main populations of São Paulo Marsh Antwren. Genotypic data for 17 microsatellite loci presented as a three-digits GENEPOP file. Missing data are reported as 000000.
(TXT)

S1 Table Summarized microsatellite data for the main populations of São Paulo Marsh Antwren. Number of analyzed individuals (*N*), number of alleles (*N*_A_), allelic richness (*A*_R_), number of private alleles (*A*_P_), allele range in base pairs (T), observed (*H*_O_) and expected (*H*_E_) heterozygosities, inbreeding coefficient (*F*_IS_), and its probability of being different from zero (*P*) found for each analyzed microsatellite locus in three populations of the São Paulo Marsh Antwren. Critical value after Bonferroni correction is 0.001.
(DOCX)
