# Supplementary material for: Marshes as “Mountain Tops”: Genetic Analyses of the Critically Endangered São Paulo Marsh Antwren (Aves: Thamnophilidae)
Source: PLoS One. 2015 Oct 8;10(10):e0140145. doi: 10.1371/journal.pone.0140145 (PMC4598188; doi:10.1371/journal.pone.0140145)
Supplement: S1 Table — Number of analyzed individuals (N), number of alleles (N A), allelic richness (A R), number of private alleles (A P), allele range in base pairs (T), observed (H O) and expected (H E) heterozygosities, inbreeding coefficient (F IS), and its probability of being different from zero (P) found for each analyzed microsatellite locus in three populations of the São Paulo Marsh Antwren. Critical value after Bonferroni correction is 0.001. (DOCX) [file pone.0140145.s002.docx]

**S1 Table. Microsatellite data for the main populations of São Paulo Marsh Antwren.** Number of analyzed individuals (*N*), number of alleles (*N*_A_), allelic richness (*A*_R_), number of private alleles (*A*_P_), allele range in base pairs (T), observed (*H*_O_) and expected (*H*_E_) heterozygosities, inbreeding coefficient (*F*_IS_), and its probability of being different from zero (*P*) found for each analyzed microsatellite locus in three populations of the São Paulo Marsh Antwren. Critical value after Bonferroni correction is 0.001.

| Population/  Analyzed locus | *N* | *N*_A_ | *A*_R_ | *A*_P_ | T (pb) | *H*_O_ | *H*_E_ | *F*_IS_ | *P* |
| --- | --- | --- | --- | --- | --- | --- | --- | --- | --- |
| Mogi das Cruzes |  |  |  |  |  |  |  |  |  |
| Fpa11 | 26 | 10 | 8.25 | 1 | 395-467 | 0.81 | 0.84 | 0.035 | 0.453 |
| Fpa13 | 26 | 3 | 2.88 | 0 | 368-393 | 0.35 | 0.36 | 0.034 | 0.525 |
| Fpa14 | 26 | 6 | 5.13 | 0 | 575-599 | 0.73 | 0.70 | -0.038 | 0.728 |
| Fpa15 | 26 | 4 | 4.00 | 0 | 216-264 | 0.77 | 0.73 | -0.052 | 0.741 |
| Fpa17 | 26 | 6 | 5.94 | 0 | 452-472 | 0.89 | 0.82 | -0.084 | 0.889 |
| Fpa18 | 26 | 6 | 5.25 | 2 | 364-388 | 0.69 | 0.77 | 0.097 | 0.233 |
| Fpa23 | 26 | 4 | 3.99 | 0 | 237-255 | 0.73 | 0.73 | -0.008 | 0.628 |
| Fpa24 | 26 | 7 | 5.83 | 1 | 429-479 | 0.65 | 0.76 | 0.142 | 0.110 |
| Fpa25 | 26 | 5 | 4.91 | 1 | 252-288 | 0.69 | 0.67 | -0.040 | 0.719 |
| MyEx19 | 26 | 5 | 4.61 | 1 | 292-313 | 0.65 | 0.62 | -0.048 | 0.729 |
| MyEx41 | 26 | 5 | 4.25 | 0 | 247-275 | 0.65 | 0.67 | 0.030 | 0.499 |
| MyEx46 | 26 | 10 | 8.69 | 4 | 298-346 | 0.85 | 0.87 | 0.026 | 0.431 |
| Mex034 | 26 | 6 | 5.48 | 0 | 251-275 | 0.81 | 0.79 | -0.019 | 0.660 |
| Mex120 | 26 | 6 | 5.47 | 0 | 223-243 | 0.89 | 0.80 | -0.115 | 0.937 |
| Mex140 | 26 | 9 | 7.49 | 3 | 196-240 | 0.58 | 0.82 | 0.299 | 0.005 |
| Mex162 | 26 | 5 | 4.38 | 0 | 232-252 | 0.60 | 0.64 | 0.103 | 0.264 |
| Mex176 | 26 | 5 | 4.37 | 1 | 206-234 | 0.58 | 0.54 | -0.064 | 0.763 |
| Salesópolis |  |  |  |  |  |  |  |  |  |
| Fpa11 | 17 | 8 | 7.28 | 2 | 383-449 | 0.77 | 0.77 | 0.002 | 0.590 |
| Fpa13 | 17 | 2 | 2.00 | 0 | 388-393 | 0.71 | 0.50 | -0.433 | 0.993 |
| Fpa14 | 17 | 6 | 5.48 | 1 | 575-595 | 0.77 | 0.67 | -0.156 | 0.930 |
| Fpa15 | 17 | 3 | 3.00 | 0 | 216-264 | 0.71 | 0.53 | -0.352 | 1.000 |
| Fpa17 | 17 | 5 | 4.71 | 0 | 456-472 | 0.41 | 0.62 | 0.347 | 0.031 |
| Fpa18 | 17 | 4 | 3.77 | 0 | 364-376 | 0.47 | 0.57 | 0.179 | 0.224 |
| Fpa23 | 17 | 5 | 4.99 | 1 | 231-255 | 0.59 | 0.75 | 0.216 | 0.083 |
| Fpa24 | 17 | 4 | 4.00 | 0 | 429-459 | 0.77 | 0.73 | -0.056 | 0.732 |
| Fpa25 | 17 | 5 | 4.76 | 0 | 260-288 | 0.41 | 0.62 | 0.343 | 0.025 |
| MyEx19 | 17 | 3 | 3.00 | 0 | 301-313 | 0.53 | 0.64 | 0.182 | 0.198 |
| MyEx41 | 17 | 5 | 4.71 | 0 | 247-263 | 0.65 | 0.63 | -0.023 | 0.667 |
| MyEx46 | 17 | 5 | 4.75 | 1 | 298-338 | 0.77 | 0.64 | -0.199 | 0.972 |
| Mex034 | 16 | 4 | 4.00 | 0 | 251-271 | 0.75 | 0.71 | -0.056 | 0.753 |
| Mex120 | 17 | 6 | 5.53 | 0 | 223-251 | 0.82 | 0.72 | -0.143 | 0.924 |
| Mex140 | 17 | 5 | 4.71 | 0 | 204-232 | 0.53 | 0.53 | -0.007 | 0.670 |
| Mex162 | 17 | 6 | 5.94 | 1 | 232-256 | 0.65 | 0.82 | 0.211 | 0.074 |
| Mex176 | 17 | 5 | 4.76 | 0 | 222-238 | 0.88 | 0.74 | -0.203 | 0.954 |
| São José dos Campos |  |  |  |  |  |  |  |  |  |
| Fpa11 | 14 | 10 | 9.85 | 3 | 371-467 | 0.86 | 0.90 | 0.049 | 0.432 |
| Fpa13 | 14 | 4 | 4.00 | 1 | 368-398 | 0.50 | 0.71 | 0.300 | 0.062 |
| Fpa14 | 14 | 6 | 5.86 | 1 | 575-603 | 0.79 | 0.77 | -0.029 | 0.698 |
| Fpa15 | 14 | 5 | 4.93 | 2 | 216-260 | 0.64 | 0.69 | 0.068 | 0.450 |
| Fpa17 | 14 | 6 | 5.86 | 1 | 452-560 | 0.79 | 0.76 | -0.032 | 0.675 |
| Fpa18 | 14 | 6 | 5.92 | 2 | 352-376 | 0.64 | 0.71 | 0.100 | 0.348 |
| Fpa23 | 14 | 4 | 3.93 | 1 | 237-261 | 0.36 | 0.43 | 0.172 | 0.280 |
| Fpa24 | 14 | 8 | 7.78 | 2 | 434-479 | 0.79 | 0.78 | -0.007 | 0.633 |
| Fpa25 | 14 | 5 | 5.00 | 1 | 268-288 | 0.64 | 0.74 | 0.140 | 0.269 |
| MyEx19 | 14 | 3 | 3.00 | 0 | 292-313 | 0.43 | 0.58 | 0.271 | 0.173 |
| MyEx41 | 14 | 5 | 4.93 | 1 | 251-275 | 0.86 | 0.74 | -0.169 | 0.936 |
| MyEx46 | 14 | 6 | 5.85 | 1 | 318-338 | 0.86 | 0.77 | -0.114 | 0.874 |
| Mex034 | 14 | 6 | 5.92 | 1 | 255-275 | 0.79 | 0.76 | -0.036 | 0.725 |
| Mex120 | 13 | 7 | 7.00 | 3 | 223-251 | 0.85 | 0.75 | -0.128 | 0.939 |
| Mex140 | 14 | 6 | 5.85 | 0 | 219-255 | 0.71 | 0.65 | -0.111 | 0.894 |
| Mex162 | 14 | 6 | 6.00 | 1 | 232-252 | 0.86 | 0.84 | -0.016 | 0.677 |
| Mex176 | 14 | 5 | 5.00 | 1 | 226-242 | 0.57 | 0.78 | 0.275 | 0.069 |
